# Major economic burden of musculoskeletal and connective tissue diseases in Slovenia: Marjetka Jelenc

**DOI:** 10.1093/eurpub/ckac130.195

**Published:** 2022-10-25

**Authors:** M Jelenc, S Sedlak, S Simonović

**Affiliations:** 1 Center for Health Care, National Institute of Public Health, Ljubljana, Slovenia; 2 Faculty of Health Sciences, University of Maribor, Maribor, Slovenia

## Abstract

**Background:**

Musculoskeletal and connective tissue diseases represent a growing public health problem, a great burden on health systems and place a heavy burden on society as a whole. Patients with musculoskeletal and connective tissue diseases often retire early, and any early retirement that could be prevented represents a loss of human capital, which means great social and economic damage to society. The present study was conducted in order to calculate for the first time in Slovenia the costs of the six most common diseases of the musculoskeletal and connective tissue diseases for the period from 2016-2018.

**Methods:**

The calculated burden of musculoskeletal and connective tissue diseases was based on the calculation of various direct and indirect costs cross-sectionally at the level of one year. The methodology of National Transfer Accounts (NTA) was used for the calculation of indirect costs. Six major diagnoses based on the International Classification of Diseases and Related Health Problems for statistical purposes were selected for calculation. Data were obtained from different national routine databases.

**Results:**

Calculations of the economic burden of musculoskeletal and connective tissue diseases in Slovenia showed that the economic costs of six selected diagnoses in the period 2016-2018 averaged about 5% of total health expenditure or 0.4% of gross domestic product in this period. The highest direct costs were hospitalizations, followed by costs for medicines, first curative visits at the primary level and visits to the outpatient clinic at the secondary level.

**Conclusions:**

The results of the first calculation of the burden of musculoskeletal and connective tissue diseases in Slovenia showed a high economic burden of these diseases in the period from 2016 to 2018. The economic burden is underestimated and would be significantly higher considering all diagnoses from this group of diseases.

**Key messages:**

- Reducing the burden of musculoskeletal and connective tissue diseases is absolutely necessary in view of the aging population and in the light of the sustainability of health systems.
- The preparation of strategic documents for the promotion of musculoskeletal health and the consistent implementation of their contents has become urgent in Slovenia.

